# Perceptions and Experiences of Parents of Burn-Injured Children during Hospital Stay: A Need for Integrated Care

**DOI:** 10.3390/healthcare12060614

**Published:** 2024-03-08

**Authors:** Martim Santos, Ana Ferraz, Maria Garcia, M. Graça Pereira

**Affiliations:** 1 Psychology Research Centre (CIPsi), School of Psychology, University of Minho, 4710-057 Braga, Portugal; martimsantos@email.com (M.S.); anasofiaferraz93@gmail.com (A.F.); 2 Department of Pediatric Surgery, Centro Hospitalar Universitário São João (CHUSJ), 4200-319 Porto, Portugal; maria.fernandez@chsj.min-saude.pt

**Keywords:** burns, children, parents, needs, psychological distress, psychosocial adjustment, hospital stay, integrated care, quality of life, qualitative design

## Abstract

Pediatric burn injuries are a critical medical condition that triggers a series of ongoing multifactorial stressors that affect both children and their families. To inform healthcare research and clinical practice, this study aimed to understand and describe the perceptions and experiences of the parents of burn-injured children during hospital stay. Forty-six parents (thirty-eight mothers) of forty-six children (eighteen girls) with a mean age of 2.28 years (*SD* = 1.52) answered ten open-ended questions. This qualitative study was conducted in a referral hospital in the northern region of Portugal. Qualitative data were analyzed using an inductive content analysis. Five key themes emerged from the data analysis: diving into the crisis of the child burn injury, being together and in good hands, becoming aware of an uncertain future, enhancing supportive care and environment, and finding ways to guide parents. Qualitative findings underlined the pressing need for integrated care within this context. Parents were significantly burdened and distressed during the inpatient phase. These parents should be included in the integrated care plan starting from admission. Understanding and addressing parents’ healthcare needs and psychosocial adjustment difficulties is paramount to the development of future intervention programs and the delivery of suitable integrated healthcare.

## 1. Introduction

Globally, burn injuries are a worrying public health problem, particularly in low- and middle-income countries [1,2], and caused almost 111,300 deaths in 2019 alone, mainly among children aged 1–4 years [3]. The World Health Organization (WHO) African and Southeast Asia regions account for almost two-thirds of all cases, and in Bangladesh, Colombia, Egypt, and Pakistan, 17% and 18% of pediatric burn injuries result in temporary and permanent disabilities, respectively [2]. Despite these worrisome data, a recent scoping systematic review focused on burn injury prevention in low- and middle-income countries concluded that political initiatives and environmental interventions (e.g., making the home environment safer for children) remain sparse [4]. Furthermore, the implementation of accessible and efficient services [5] and prevention programs are crucial [6] since burn injuries are a leading cause of preventable mortality and morbidity in childhood worldwide [2], especially in preschool age [7], with a peak prevalence during the first year of life [8]. In fact, there is a higher incidence of burn injuries in children than in adults [9], and in early childhood, boys are at greater risk than girls [7]. Scalds and contact burns are among the most recurrent causes of burn injuries, preponderantly affecting children under five years of age, on the upper torso, arms, face, and hands [8]. Although advances in medicine and healthcare have contributed to improving overall clinical outcomes and, subsequently, decreasing hospital stays and mortality rates [1,3], burns remain one of the most traumatic injuries that a child and their family may experience [10].

Pediatric burn injuries generally require long hospital stays and acute critical care when compared to other non-fatal childhood injuries [11], representing a substantial economic burden, particularly in low-income areas [5]. A systematic review focused on the costs of burn care concluded that the mean total healthcare costs in higher-income countries are over 88,000 dollars per patient, ranging from 704 to 717,306 dollars, with a median of 44,024 dollars, while in low- and middle-income countries, the mean total was 5196 dollars, ranging from 102 to 15,555 dollars, with a median of 3559 dollars [12]. Furthermore, there is a worldwide trend toward an increase in the number of new cases [3], which raises several healthcare concerns, particularly in countries where health systems are more vulnerable [1,2,5]. Portugal is following this trend, with 20% of the total cases being children under five years of age [13], with a median length of hospital stay of nine days, and higher average hospital costs than other countries (around 3073 euros) [14]. Despite this scenario, few studies on pediatric burns were conducted in Portugal, with the existing studies being mostly retrospective observational and focused on demographic and clinical burden indicators, e.g., [13,14]. Santos et al. [14] also emphasize the need for future studies to inform healthcare services and policies, reducing related costs and thereby establishing a pediatric burn center with differentiated and specialized evidence-based services.

More broadly, and beyond economic and epidemiological issues, burn injuries have health (e.g., delayed wound healing), physical (e.g., long-term disability and disfigurement from contractures and scarring), psychological (e.g., psychological morbidity and traumatic symptoms), and social (e.g., troubled social competence) related consequences that impair the short- and long-term quality of life of pediatric patients and their families [8,9,15,16,17,18]. Notwithstanding, a growing body of literature has shown that parents of burn-injured children experience a series of ongoing maladaptive responses, including significant emotional distress [19], feelings of guilt, blame, and shame [19,20,21], clinical symptoms of psychological morbidity, such as anxiety and depression [22,23], and traumatic reactions [20,22,24,25,26]. During the first month post-burn, some parents (approximately 25%) reported symptoms that potentially indicate posttraumatic stress disorder (PTSD) [22]. However, it is well documented in the literature that most of these symptoms tend to decrease over time [22,23,24], and hospital stay can be an opportunity for early screening and prevention, e.g., [27], which should target all (nuclear) family members [25]. Thus, addressing and understanding parents’ mental health needs and subjective appraisals of the child burn injury during hospital stay is expected to strengthen our knowledge of parents’ potentially maladaptive responses and help to prevent their emotional and psychosocial adjustment difficulties through the design of interventions tailored to the political, economic, and sociocultural context.

Prolonged hospital stays have been significantly associated with higher parents’ posttraumatic stress symptoms (PTSS) [20] and PTSD [25]. Also, the hospital environment exacerbates psychological maladaptive responses, namely maternal distress, which suggests that mothers, who are typically the child’s primary caregiver during the hospital journey, seem to be a particularly vulnerable group due to the severity of the symptoms presented [10,28]. A recent qualitative study by Karahan et al. [28] found that mothers of pediatric burn patients faced severe physical and emotional distress from the burn occurrence and throughout the inpatient phase, as a result mainly of a lack of adaptive internal coping resources and professional support. In addition, a study focusing on changes in the outcomes of caregivers of burn survivors within one-year post-burn found that increased length of hospital stays contributed to a decrease in the caregivers’ quality of life [29]. Therefore, parents’ distress threatens caregivers’ adjustment and leads them to experience more difficulties that may negatively impact the act of caring (e.g., meeting the child’s needs) [27,30]. The literature also shows significant changes in parental roles motivated by the unexpected demands of the hospital stay [26], such as remaining constantly vigilant to meet children’s needs, which also impaired the parents’ physical health (e.g., poor sleep quality and weight loss) [31]. Hence, parents play a key role during (and after) the inpatient phase [32], given the child’s age/stage of development and the type of treatment planned (e.g., long-term rehabilitation), making it essential to include parents in an integrated care plan that also promotes their engagement [29]. However, in the field of pediatric burn injuries, knowledge about parents’ perceptions and experiences regarding hospital stay is scarce [28,30,31,32,33,34,35], limiting the development of research-informed intervention care plans. A recent scoping systematic review highlighted the paucity of qualitative data on parents’ issues and concerns regarding their children’s hospital stay period [26]. Given the prevalence of pediatric burn injuries and their often prolonged hospitalization [13,14], this knowledge is paramount to achieve an in-depth understanding of the complexity of the phenomenon in order to develop effective family-centered intervention programs. 

A previous qualitative study conducted six months post-burn identified three phases that describe the parents’ journey from their perspective: experiencing the accident, the inpatient phase, and returning to the community [35]. The inpatient phase was the most challenging for parents, including, particulary, witnessing the (invasive and traumatic) medical procedures, which generated significant parental distress and were described as the “worst aspect” of the whole journey [35]. Brown et al. [33] also found that during the child’s medical procedures, parents often showed multiple indicators of emotional distress (e.g., fear, uncertainty, and concerns about the potential for permanent scarring) and traumatic reactions (e.g., leaving the room while dressings were being changed). In contrast, a recent qualitative study focusing on parents’ memories and appraisals of burn-injured children found that parents’ highly emotional memories related to the inpatient phase (e.g., children’s procedural pain) were not perceived as potentially intrusive [36]. Another qualitative study on parental presence or absence during child wound care corroborates these findings and suggests that parental participation in medical procedures produces a “sense of control”, which can mitigate its underlying traumatic nature [34]. In addition, a more recent qualitative study pointed out that meeting parents’ support needs during the hospital period (e.g., feeling cared for and having time to self-care) maximizes parents’ contribution to the child’s recovery and the maintenance of effective care [30]. In fact, the hospital stay period is considered a critical phase [31] but also an important opportunity to address and guide the provision of psychological care to parents following their child’s critical injuries [27]. 

Given all these indicators, the purpose of the present study was to study, understand, and describe the perceptions and experiences of parents of preschool children who had sustained an unintentional burn injury, during their hospital stay. This is the first study carried out in Portugal that also seeks to fill significant gaps in the literature (e.g., lack of evidence on parents’ perceptions and experiences during the inpatient phase for very young burn-injured children), thereby informing future clinical practices and the delivery of suitable integrated healthcare.

## 2. Materials and Methods

### 2.1. Design

This qualitative study is part of a larger longitudinal research project on the phenomenon of pediatric burn injuries in early childhood (0–6 years) from a biopsychosocial perspective, including medical, parental, and family environmental factors regarding the child’s health outcomes. The current study was therefore designed to target parents of preschool children who had sustained an unintentional burn injury in order to capture their subjective experiences and perceptions during the hospital stay period. A descriptive qualitative approach was adopted, based on ontological, philosophical, and epistemological principles applied to health research, in order to obtain a more comprehensive understanding and an accurate description of the participant’s experience and the meanings they attribute to the phenomenon under study [37]. Furthermore, this approach is considered the most appropriate when little is known about a particular topic [38], and the goal is to gather information to serve as an empirical basis for informing public policy and developing future evidence-based interventions [39].

### 2.2. Research Team

M.S. (male) is a clinical and health psychologist and a PhD student in Applied Psychology, working on his doctoral thesis on this topic, with training in qualitative methods. A.F. (female) is also a clinical and health psychologist and a PhD student in Applied Psychology with training in qualitative methods. M.G. (female) has been a graduate hospital assistant in pediatric surgery since 2014 and is responsible for the Pediatric Burns Unit at the hospital. M.G.P. (female) is an associate professor with habilitation, a clinical and health psychologist/psychotherapist, with three decades of experience in supervising and conducting qualitative and quantitative research on chronic illness and health promotion. 

### 2.3. Setting

The Pediatric Surgery Department, part of the Autonomous Management Unit of Women and Children (UAG MC), provides surgical care to pediatric patients up to 18 years with different medical conditions and needs (e.g., congenital anomalies, injuries, oncological problems) and supports neonatal/pediatric intermediate and intensive care units. The department has 18 inpatient beds (maximum two patients per room) and includes a recent Pediatric Burns Unit with five individual rooms and its operating room. 

Since 2021, a playroom (i.e., Ronald McDonald Playroom) has been available for pediatric patients and their parents, with many play resources/activities and seven distinct areas (e.g., recreational, social, and multi-sensory relaxation area). The establishment of the Ronald McDonald Playroom, based on a family-centered care model, aims to promote the emotional well-being of both families and children during the inpatient phase. Only one of the parents/legal guardians may be with the child during hospital stay without (visiting hours) restrictions. 

### 2.4. Participants and Procedure

Eligible participants were parents/legal guardians who met the following inclusion criteria: (a) 18 years or older; (b) Have a child aged between one month and six years who recently sustained an unintentional burn injury that required hospital admission; (c) Accompany the child during hospital stay and subsequent treatment. Exclusion criteria comprised insufficient language proficiency to complete the study protocol, psychiatric or oncological diagnosis in the child’s medical records, and/or the suspicion that the burn injury was the result of abuse or neglect.

Potential eligible parents were identified, addressed, and invited to participate in the current study by a specialist pediatric surgeon (M.G.) during clinical rounds in the Pediatric Surgery Department of a referral hospital in the Northern region of Portugal. Recent national data indicate that 43.9% of burn admissions (the vast majority) were recorded in the northern region of mainland Portugal [14], which justifies the decision to carry out this study in this geographical area.

All parents who agreed to take part in this study were informed by M.S. of the aims of the study, confidentiality, anonymity, and its voluntary nature. Thereafter, the parents/legal guardians gave written informed consent for their and their child’s participation. 

Data collection took place between March 2021 and May 2023, and the study protocol was implemented by M.S. during the child’s stay in the hospital’s Pediatric Surgery Department that ranged between one and seventeen days (*M* = 6.26, *SD* = 3.09) after the occurrence of the burn event. After completing part A of the study protocol, which included sociodemographic and clinical questionnaires (e.g., gender, age, how the burn event occurred), parents filled out a “logbook” with 10 open-ended questions (part B) to gather qualitative data and returned it prior to the child’s discharge. Parents were asked to reflect and honestly write about their experience and their child’s experience during the hospital stay to help benefit other children and their families to have a better experience, in the future. The questions included in the logbook were developed by our multidisciplinary research team (i.e., specialists in clinical and health psychology, pediatric trauma, and pediatric surgery) and covered many key issues (e.g., perceived needs and adjustment difficulties) supported by the available burn-specific literature on the topic, e.g., [19,30], in order to capture the subjective experiences and perspectives of parents regarding the hospital stay period. Thus, the following open-ended questions were presented: (i) *During the hospital stay, I think my child felt…*; (ii) *During my child’s hospital stay, I felt…*; (iii) *In my opinion, the most positive situation for my child during the hospital stay was…*; (iv) *In my opinion, the most negative situation for my child during the hospital stay was…*; (v) *My biggest concern about the future is that my child…*; (vi) *The fact that my child was hospitalized during the COVID-19 pandemic left me…*; (vii) *The main difficulties I experienced during my child’s hospital stay were…*; (viii) *The main difficulties my child experienced during the hospital stay were…*; (ix) *I think my child would feel better in hospital if…*; (x) *If I could leave a message to other parents who might find themselves in this situation in the future, I would say…* A blank box was also added for other relevant comments, reflections, and/or recommendations. The study protocol (parts A and B) took around 30 min to complete. The child’s clinical data were recorded by the specialist pediatric surgeon (M.G.), who had no role in data collection, analysis, and interpretation.

### 2.5. Ethics

This study was carried out following the guidelines of the Declaration of Helsinki [40] and was approved by the Ethics Committee for Research in Social and Human Sciences of the Ethics Council of the University of Minho (CEICSH 020/2020) and the Ethics Committee of the hospital involved (CE 429/20).

### 2.6. Data Analysis

Qualitative data were duly anonymized (to ensure confidentiality), imported into QSR International’s NVivo 10 software, and then analyzed in the same order as it was carried out, using content analysis [41], aligned with the post-positivist paradigm in which the authors are positioned [42]. Bardin [41] established the following sequential steps for the proper organization and data analysis: pre-analysis, exploration of data, and treatment of the results (including inference and interpretation). After carefully reading the participants’ records and an overview of the data, two trained researchers/coders (M.S. and A.F.) independently organized/mapped the relevant information and highlighted the key points to identify codes. The codes that emerged from the data were identified, and any differences were resolved after discussion and consensus, resulting in a draft codebook. An experienced researcher and supervisor (M.G.P.) reviewed, discussed, and helped refine the codes to obtain a final codebook. Themes and sub-themes were independently selected, following an iterative inductive process, and differences were again discussed and resolved through consensus. To strengthen the trustworthiness and consistency of the findings, Goodell et al.’s [43] recommendations (e.g., practice applying the codebook together and compare and discuss the coding process) were followed. The data were then analyzed, reorganized, and interpreted. M.S. coded all the data, and A.F. coded approximately 35% (16 records) independently. Inter-rater agreement, using the Kappa Statistic, was 0.82, which is considered almost perfect [44]. Data saturation was reached with 46 participants, as no new information emerged during the coding process, and informational redundancy was verified [45]. Finally, descriptive statistics were performed to describe the sociodemographic and clinical characteristics of the participants, using IBM SPSS Statistics (Statistical Package for the Social Sciences) version 29.

## 3. Results

### 3.1. Participants Characteristics

Forty-six parents (38 mothers), with an average age of 33.54 years (*SD* = 5.82), of forty-six burn-injured children (18 girls), aged between eight months and six years (*M* = 2.28, *SD* = 1.52) took part in this qualitative study (Table 1 and Table 2). The majority of children (78.3%) had never been hospitalized before. Of the burn injuries that occurred at home, the majority were in the kitchen (69.6%) and the living room (15.2%). The number of burn regions sustained ranged from 1 to 5 (*M* = 2.28, *SD* = 1.11), with the upper limbs (forearm, hands, and arm, 39.1%, 37%, and 28.3%, respectively) and the face (23.9%), being the most affected areas. All children received pain treatment, and among the planned skin grafts, synthetic skin grafts (52.2%) and partial skin grafts (32.6%) were the most common.

### 3.2. Themes

Five key themes emerged from the qualitative analysis: (1) Diving into the crisis of the child burn injury; (2) Being together and in good hands; (3) Becoming aware of an uncertain future; (4) Enhancing supportive care and environment; (5) Finding ways to guide parents. All themes except one (i.e., enhancing supportive care and environment) represented 93.48% or more (*n* ≥ 43) of the total cases. Table 3 provides an overview of the themes and sub-themes (generated within the themes), as well as sub-themes distribution (frequency) and representative quotes (in quotation marks) from the parents’ records. 

### 3.3. Diving into the Crisis of the Child Burn Injury

Hospital stay forces parents to confront the seriousness of their child’s burn injury and raises new and unexpected challenges that threaten the adjustment of both parents and patients. Within this theme, five sub-themes were identified: negative trauma responses, facing medical procedures, changes in the child’s functioning, COVID-19 stressors, and unmet needs.

#### 3.3.1. Negative Trauma Responses

Thirty-seven parents experienced at least one maladaptive reaction during the inpatient phase, as well as negative psychological, cognitive, emotional, and physical responses (e.g., self/other blame attribution, impotence, physical and emotional exhaustion), which encompassed normative and potentially adaptive affective responses to their child’s burn injury (e.g., worries, negative mood). Most of these parents reported feeling “distressed”, “exhausted”, “sad”, “worried”, “anxious”, and “guilty”. In addition, intrusive memories related to the burn event were mentioned as a factor exacerbating their distress and maladjustment: “*I felt very bad. Distressed, worried, completely exhausted! I can’t sleep, because even if I close my eyes, everything is very fresh in my memory. Sometimes I find myself acting as if I were there at that moment (when the accident occurred). I can’t think about anything else and being locked up in here doesn’t help*.” (mother of a three-year-old boy). In fact, the parents who witnessed the burn accident reacted more negatively to the whole process and described a greater variety of traumatic reactions (e.g., avoidance), and for one of them, this situation reactivated similar traumatic memories: “*(…) it makes me relive what I’ve been through because I also got burned with hot milk around this time*.” (mother of a two-year-old boy). Also, in the early stages of hospital stay, burn-injured children displayed negative psychological and emotional reactions, mainly due to the “unfamiliar environment” and seeing “strange people”. This whole new environment initially caused “fear”, “anger”, “restlessness”, and a “feeling of being trapped” in many patients, which dissipated over time: “*My son felt very trapped because he was used to playing outdoors, with the animals, with his brother and cousin, getting fresh air, and that really affected him. (…) then he adapted to the hospital and the people, but at first, it was very difficult to calm him down! He was very restless and scared*.” (mother of a one-year-old boy).

#### 3.3.2. Facing Medical Procedures

Thirty-four parents described medical procedures as a traumatic experience, both for the children and for themselves. Spending long days in the hospital and witnessing the child’s suffering due to invasive and painful treatments was considered “one of the most devastating experiences” for parents during this phase and “completely traumatizing for the children”. One mother even stressed that: “*The most difficult time was the dressing changes*.” (mother of one-year-old boy).

Dressing changes were the procedure most often mentioned as “the most negative aspect” and the main factor contributing to the child’s “pain”. For example: “*He never seemed to have any pain or discomfort, only when it was time to change the dressings*.” (mother of a one-month-old boy). Less recurrently, the “incision to place the catheter” was also mentioned as “painful” and “traumatic” for the child. Furthermore, some parents explained that any element the child associated with taking medication or treatments (e.g., the operating room; conditioned stimulus) was enough for them to become reactive and start crying (conditioned response): “*(…) whenever someone approached her, she would start crying because she thought they were going to change her bandages or force her to take medication*.” (mother of a one-year-old girl).

#### 3.3.3. Changes in the Child’s Functioning

More than half of the parents reported that observing differences in their child’s baseline functioning (compared to the period before sustaining a burn injury) was a source of distress. In addition to the significant changes in the child’s routines, this situation has led to some developmental setbacks and impaired functionality. Limitations to a child’s mobility were the difficulties most emphasized by parents. For example: “*He can’t even walk because he has to be stuck to this machine. If only I could walk with him a little bit, that would be better…*” (mother of a two-year-old boy); and “*(…) It hurts me a lot to see him wanting to crawl and not being able to*.” (father of a one-year-old boy). Other important changes in health-related domains of the child’s functioning were also reported, including appetite (e.g., loss of appetite, refusal to eat), skin (e.g., itching), and sleep problems (e.g., could not sleep a whole night, nightmares). Specifically, one mother attempted to describe the content of the nightmares: “*It seems like he dreams about what’s going on during the dressing changes and wakes up scared*.” (mother of a two-year-old boy). As a result of these changes, the child’s autonomy was compromised, making them even more dependent on their caregivers and increasing the parents’ burden: “*(…) he can’t go out because of the catheter in his foot. I have to be dedicated to him at all times because he is completely dependent on me; he already was, but now he is even more so*.” (father of a one-year-old boy).

#### 3.3.4. COVID-19 Stressors

Despite this study covering different waves of the COVID-19 pandemic, the impact of COVID-19 seemed to be relatively minor for most parents. Nevertheless, parents’ answers were divided into two different groups regarding COVID-19-related stressors at a time when (a) restrictions were more severe and (b) restrictions were relaxed or lifted. Regarding the first group, parents expressed “concern” and “fear” since a positive test would mean delaying planned treatments: “*(…) because the first test (of the child) was inconclusive and that delayed the whole process. Consequently, he couldn’t receive the care he should have been receiving*.” (mother of a one-year-old boy). Notwithstanding, parents considered that the major obstacle during this period was social restrictions. On one hand, visits were also limited, burdening the caregiver who accompanied the child: “*I have to carry all this on my own because I can’t exchange with my husband, or receive visits from my family*” (mother of a two-year-old girl); on the other hand, carrying out tests entailed more costs: “*(…) for visits to take place, a test has to be carried out which, after four tests, is very expensive*.” (mother of a five-year-old girl). To get around these constraints, parents resorted to technology (e.g., video calls); however, they stated that nothing replaces physical contact at such a critical time: “*I am always on video call, but it is not the same. I need to feel them, touch them (nuclear family members)…*” (mother of a two-year-old boy). As for the last group, parents reported minimal impact, although they had to wear masks. Interestingly, for some parents, COVID-19 has increased their “sense of safety and security” in the hospital: “*(…) because there is more attention to everything and things are cleaner.*” (mother of a one-year-old boy). In general, although at an early stage, COVID-19-related stressors contributed to increased difficulties in the treatment and adaptation process due to protocol issues and social restrictions, respectively; of the thirty-three parents who identified a negative influence of the pandemic during hospital stay, the majority described it as a minimal to a small influence: “*I am so focused on other things that this completely passes me by. In a way, it gets in the way a little, because I can’t be with my other two kids, but this whole situation and what it entails is already so bad that COVID is the least of our problems*.” (mother of a one-year-old boy).

#### 3.3.5. Unmet Needs

Thirty-eight parents shared common unmet needs (their own and those of their children) covering various dimensions, including psychosocial, emotional, and physical needs. Regarding parents’ unmet needs, lack of support from the hospital staff was the most frequently reported issue, followed by the lack of family/social support. Also, some parents reported difficulties in meeting their child’s needs due to a lack of support from the hospital staff (e.g., having to carry the child all the time and having no one to support them, bathing the child, feeding the child). Most parents felt “neglected” and believed that the hospital staff should have given them more support and resources to cope with the different challenges involved in the inpatient phase (e.g., informational) in order to reduce their discomfort and burden. Several parents even mentioned they had lost their identity and felt reduced to “the child’s companion”: “*I am just the mother. I don’t have a name here, I am just my daughter’s legal guardian. She is the patient. That is how they treat me*.” (mother of a one-year-old girl). For some parents, these attitudes remained evident in many circumstances, namely “having to sleep in a recliner chair”, which was described as “extremely uncomfortable”. One mother also added: “*I feel that there is a huge lack of support and humanization of care. Here we are treated only as “the parents”. My son is completely dependent, he is only one year old, and they do not help me to go out to buy food. My immune system has fallen, and I am breastfeeding, which is a risk for me and my son. This system is not at all “friendly” to us (parents). It is unthinkable to sleep in this armchair for nights on end with a child on your lap*.” (mother of a one-year-old boy). Most parents pointed out that sleeping in a recliner chair affected their sleep and their physical and psychological well-being. Parents also identified a lack of opportunities for self-care. For example, some parents also stressed that they needed more time for themselves and more autonomy (e.g., going outside). These aspects were more relevant for parents with some physical conditions, such as pregnancy or chronic illness: “*I am pregnant and I sleep in a recliner chair, which is not comfortable at all! It does not allow me to sleep well and causes me a lot of back pain*.” (mother of a two-year-old boy). Regarding children’s needs, parents identified needs related to homesickness and family. For example: “*(…) he really missed playing with his cousins and friends, especially missing his mother and grandparents. Basically, homesickness*.” (father of a three-year-old boy). 

### 3.4. Being Together and in Good Hands

The crisis imposed a “break from reality”, which was an opportunity to strengthen parent-child ties, making them a unit capable of facing this challenge together. At the same time, trust in the healthcare team and the medical treatment received, as well as being engaged throughout the process, seemed to have been key factors in mitigating the side effects of the initial shock. Two sub-themes were identified: child’s well-being and recovery and quality of healthcare and environment.

#### 3.4.1. Child’s Well-Being and Recovery

Thirty-four parents pointed out that objectively monitoring their child’s recovery was a critical factor in feeling better and more confident, suggesting that as their child improved, the parents also improved. For these parents, the child’s recovery was closely linked to the well-being of both of them, particularly the child: “*(…) when they removed the catheter from one of his hands. He was very happy, he even said ‘We are about to go home’*.” (mother of a two-year-old boy). Thus, more than half of the parents mentioned that “the most positive aspect” of this process was the “child’s observable recovery”, regardless of whether it was progressive or minimal: “*When my daughter was able to eat again, it was one of the happiest days of my life*.” (mother of a ten-month-old girl). Therefore, parents considered that their presence and participation in all the procedures was important in maintaining the child’s well-being, which overall was described as positive: “*My son is adjusting well and, as he recovers, his mood and social interaction improve a lot, especially with the other children (pediatric inpatients) (…) Being here with me helped him feel more secure*.” (mother of an eight-month-old boy).

#### 3.4.2. Quality of Healthcare and Environment

Parents seemed to have gradually adapted to the hospital environment, accepting and integrating the whole event, as a result of the combination of several protective factors they attributed essentially to the quality of the healthcare, including the resources and activities to distract/entertain (e.g., clown doctors) their children, and the possibility of interacting with other parents of patients with the same/similar medical conditions. More than half of parents considered that the healthcare team provided all the necessary care for their child’s well-being and recovery: “*My son was always very well cared for and embraced by everyone. All the staff, nurses, doctors, and assistants, are caring and attentive to the children. They are in good hands*!” (mother of a six-year-old boy). Of the thirty-five parents who identified issues within this subtheme, the majority mentioned the “playroom” as the aspect most appreciated by their children: “*(…) Everything is new in the playroom, it is full of toys. She gets excited and wants to touch everything! She just wants to be there*.” (mother of a one-year-old girl). 

Regarding parents, sharing experiences with other parents helped to assimilate the whole experience and minimize the feeling that it only happened to them: “*Listening to other parents’ stories was important for me to realize that this did not just happen to me. Also, those who have been here longer have helped me deal with it all*!” (father of a three-year-old girl).

### 3.5. Becoming Aware of an Uncertain Future

As the child recovers, other questions usually arise related to the post-discharge phase, namely the short- and long-term effects of the burn injury. Within this theme, two sub-themes were identified: ongoing suffering of the child and (potential) permanent scaring, and concerns about being blamed and that a burn might happen again.

#### 3.5.1. Ongoing Suffering of the Child and (Potential) Permanent Scarring

Forty-one parents expressed concern that their children would experience some kind of physical or psychological sequelae as a result of the burn injury. Most parents reported concerns about the wound healing process, with some parents emphasizing their “fear of permanent visible scars”. Less frequently, parents also highlighted potential functional constraints that could condition the child’s normative (physical) development. A mother who also sustained a burn injury in childhood pointed out the following: “*I know what he will suffer later at school, in social groups, and other contexts, because I went through the same thing*.” (mother of a two-year-old boy). In fact, the return to kindergarten and/or the community was anticipated by some parents as potentially threatening to their children. For example: “*When he goes back to kindergarten, how will the other children react? I am afraid they will ask him what happened to his face or bully him*.” (mother of a three-year-old boy). Other parents mentioned that they were afraid that there would be “episodes of bullying” and “social exclusion”. In addition, they fear that the child will develop traumatic reactions or other maladaptive psychological outcomes, including “low self-esteem” and “denial of the situation”. For some parents, the challenge was dealing with uncertainty: “*(…) Everything is open, he may not have any physical marks if the skin grafts go well, but the psychological ones, I don’t know… this uncertainty destroys me*.” (father of a one-year-old boy).

#### 3.5.2. Concerns about Being Blamed and That a Burn Might Happen Again

Six parents reported being worried that their children would blame them in the future. Interestingly, these parents were the same ones who reported feelings of guilt and expressed perceptions of being judged by others. For example: “*(…) I am afraid that he will blame me or think that this was intentional*.” (mother of a two-year-old boy). In addition, a few parents stated that their biggest concern about the future was that their child would suffer a burn injury again. For example: “*(…) the possibility of my son being burned again*.” (mother of a one-year-old boy). These parents were aware that this type of accident was unpredictable and uncontrollable, showing threatening risk perceptions: “*(…) If this happened at home, under our supervision, it could happen anywhere, at any time*.” (mother of a two-year-old girl).

### 3.6. Enhancing Supportive Care and Environment

Thirty-five parents considered that there were controllable factors that could promote a better inpatient experience, and they intersected with some of the needs and difficulties they listed. These factors included strengthening support and adapting the care plan, as well as the improvement of hospital facilities. The remaining parents, who reported being generally satisfied with the hospital’s services and facilities, did not mention any aspects for improvement.

#### 3.6.1. Strengthening Support and Adapting the Care Plan

As parents were an essential part of the child’s recovery, given that their children were in a situation of increased vulnerability (due to their clinical condition, but above all due to their stage of development, which makes them more dependent on their parents), more than half of the parents expected to find a more supportive and humanized service, responsive to their needs and difficulties. Among the several suggestions, most parents focused on increasing the number and frequency of visits, followed by the need to receive more support in caring for the child (e.g., help with meals), as well as for themselves to be able to “get back on their feet”. For example: “*It was essential for me and my son that they let the godparents and grandparents in*.” (mother of a six-year-old boy); and “*Not having support took away a lot of my energy to look after him and if I was well, he would be better off, because I would be able to devote more of myself to him*.” (mother of a one-year-old boy). Some parents mentioned that they had never been asked about their emotions or how they were feeling and claimed to be unaware of the existence of psychological services: “*I felt very bad and was never asked if I needed psychological support. I don’t even know if there are psychologists here. I think they should improve the services in this area*.” (mother of an eight-month-old boy). Furthermore, few parents reported that it would be important to adjust the feeding schedule to each child’s routine to help them adapt: “*(…) adjusting meal schedules for young children. They bring lunch at 1 pm and dinner at 6:30 pm, and of course he finds it strange*.” (mother of a two-year-old boy).

#### 3.6.2. Improvement of Hospital Facilities

Eight parents suggested several improvements over the existing hospital facilities, related mostly to the comfort of those accompanying the child, such as sleeping close to/in the same room as the child in “decent conditions” (sleeping in a bed) while also wishing for a single room to ensure their/their family (visitors) “privacy” (not sharing with other patients). For example: “*Everything is at the service of the child, but they forget the parents. It is important to invest in our comfort, for example, that all the rooms have bathrooms, single rooms, etc*.” (mother of a four-year-old girl). Two parents also stressed that it would be important to “better equip the social area” and “re-evaluate the cleaning conditions”.

### 3.7. Finding Ways to Guide Parents

Reflecting on their experience, most parents tried to outline a set of strategies and advice to guide other parents who might experience this situation in the future. Within this theme, three sub-themes were identified: maintaining a positive mindset and activating coping resources, recognizing parental distress, promoting the child’s well-being, and raising awareness.

#### 3.7.1. Maintaining a Positive Mindset and Activating Coping Resources

More than half of the parents mentioned the importance of maintaining a positive mindset and activating coping resources (including support resources) in order to self-regulate, stabilize, and ultimately benefit from a more adaptive inpatient experience. These parents listed a set of strategies that involved self-regulation and self-soothing functioning, “hope”, “reliance”, “faith”, and “resilience”. Regarding resilience, one mother even emphasized that “*This experience made me realize that I am more resilient than I thought.*” (mother of a three-year-old boy). Curiously, there were paradoxical reports from parents when it came to counteracting feelings of guilt. For example: “*It feels strange to say this since I am not doing it, but try not to blame yourself.*” (mother of a one-year-old girl). In this line, other parents highlighted that “*Don’t feel guilty. This is the first thing you should do. Express your feelings without fear, it relieves you.*” (father of a one-year-old boy). Six parents mentioned it was important to seek out and use all available types of support (e.g., informational, social, family) to minimize the effects of distress, better understand the process and subsequent phases (e.g., how they can help the child recover faster), and overall promote a better adjustment to the whole experience. For example: “*Seek all the help you need at the hospital, there are many alternatives available.*” (mother of a five-year-old boy). Family support was the most mentioned type of support: “*Seek the support of your family. That is the greatest support you can receive*.” (mother of a one-year-old boy). For a few parents, the development of a sense of cohesion with the child and the hospital staff is also crucial to dealing positively with this challenge: “*Together we really win. You have to trust the medical team. Trust and unity give us peace of mind and a good mood to get through this stage*.” (mother of a six-year-old boy). Even though few parents experienced the hospital stay as a “hard” and “long-lasting” process, “*A day here feels like several days*.” (mother of a ten-month-old girl), they encourage other parents to “give it time” as recovery takes time. 

#### 3.7.2. Recognizing Parental Distress and Promoting Child’s Well-Being

Of the nine parents who reflected on the importance of recognizing their distress, the majority reported that their children were well-adapted most of the time, although they experienced multiple indicators of distress. For example: “*Indeed, we are the ones who suffer the most, they may not even remember what happened. For us, it is a (psychological) scar for life*.” (mother of a one-year-old boy). For some parents, recognizing their symptoms is a key to normalize parents’ emotional states and raise awareness among healthcare providers for the need to develop a targeted care system, helping parents be fully active in their child’s therapeutic plan, particularly in maintaining care at all stages of treatment (e.g., post-discharge): “*Validate your distress, it is normal given the situation. Nobody likes to see their children in this situation*.” (mother of a six-year-old girl). In addition, twelve parents focused on the child’s well-being. These parents emphasized the child’s need to have them physically and psychologically available to meet all their needs and ensure their well-being. In addition to the dependency issues justified by the child’s stage of development, children tend to be influenced by their parents’ emotional states and behaviors (e.g., mimicking facial expressions), which leads these parents to assume that they have to “stay strong” for their children. One father even mentioned: “*Stay strong to help the child get through the situation. They feel everything we feel and depend on us a lot!*” (father of a one-year-old boy). “Our love helps them to recover” and “devoting full attention to their behavior and needs” were some of the examples most often mentioned by parents. Furthermore, one mother highlighted the importance of the parent’s support: “*Do your best for your children. They need to feel that they have the support of their parents and family*.” (mother of a one-year-old boy).

#### 3.7.3. Raising Awareness

Some parents reported not having enough information about the type of accident that happened to their child (e.g., prevalence and incidence, burn mechanisms), which posed several threats to their initial adjustment and their ability to respond effectively: “*When the accident happened, I found myself at a loss. Did I make the burn worse? I didn’t know about it or how to proceed*.” (mother of a one-year-old girl). Thus, nine parents believe that raising awareness of burn injuries is fundamental to prevent, minimize, and adaptively manage the impact of the burn event. “Accidents happen” and “it is not in our control” were the most frequent answers. For these parents, accepting the uncontrollable and unpredictable nature of the accident is imperative: “*One minute costs a life! One minute costs a life! This can happen at any time and all it takes is an instant! And there is nothing we can do about it*.” (mother of a two-year-old boy).

### 3.8. Summary of the Results and Goals for Intervention

**Figure 1:**
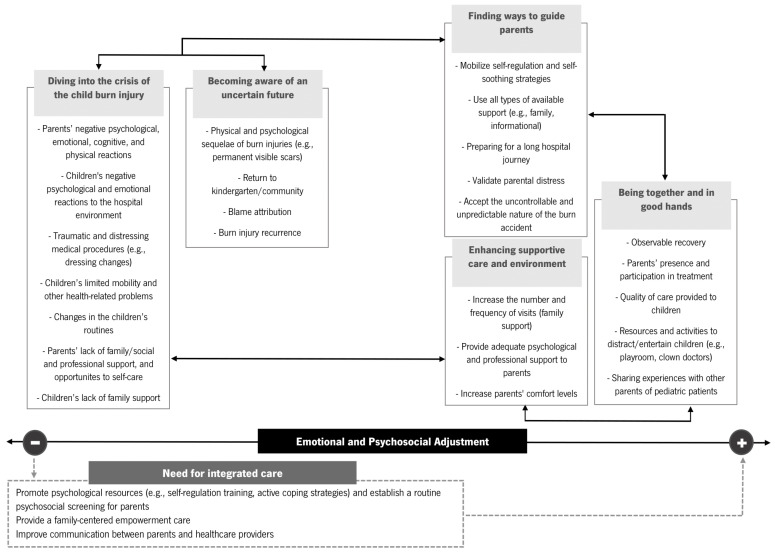
Graphical map of the main results. Theme boxes closer to one of the poles of emotional and psychosocial adjustment represent more adaptive (+) or more maladaptive adjustment (−). Bold arrows indicate the interaction between themes. Dotted-line box reflects the role of healthcare providers.

A graphical map of the main findings was designed to facilitate readers’ understanding and help guide future clinical practices and health promotion (Figure 1).

## 4. Discussion

The present study describes the qualitative findings of the parents’ perceptions and experiences regarding their burn-injured children aged between eight months and six years, during the hospital stay period. This qualitative study provides a deeper understanding of how parents and their children adjusted to the inpatient phase from the parents’ perspective and raises several implications for healthcare research and clinical practice. Parents face a highly adverse situation triggered by a primary stressor (burn event) preceded by a secondary one (dealing with the hospital experience). In addition, they are responsible for a child who, due to their age/stage of development and (critical) medical condition, is even more dependent on them. For these reasons, parents are plunged into a crisis marked by several threats to their psychological adjustment. The main findings of the current study revealed that parents were significantly burdened and distressed during the inpatient phase. Parents who witnessed the burn accident experienced intense negative emotional and psychological responses, such as psychological morbidity and feelings of guilt. These responses were also reported by McGarry et al. [35], suggesting that witnessing the burn event is a potentially traumatic experience, but due to the initial shock, parents tend to suppress their emotions. A qualitative study by Ravindran et al. [31] found similar results and highlighted that the hospital stay period was described as traumatic by all the participants and that the parental emotional and physical distress was directly related to the experience of watching their child suffer. According to the authors, parents also neglected their own needs (e.g., restricting their emotional expression with healthcare professionals) in order to focus exclusively on meeting their child’s needs [31]. In fact, recent evidence suggests that being responsive to the child’s needs is a crucial task underlying the parental role during burn wound care [34]. Thus, as expected, parents put their children’s needs before their own, although they perceive less severe maladaptive emotional and psychological reactions in their child compared to their own. Furthermore, parents seem to minimize and disguise what they are feeling, trying to show that they are “still strong” and capable of promoting their children’s physical and psychological well-being. Karahan et al. [28] found similar results in a qualitative study focused exclusively on the experiences of mothers in a pediatric burn intensive care unit and emphasized the importance of using adaptive coping strategies during this period. In addition to difficulties in emotional expression and regulation, these parents have intrusive memories of the burn accident, which threatens their psychological adjustment. In a qualitative study focused on parents’ memories and appraisals, within three to six months post-burn, parents revealed intrusive memories regarding the burn injury and subsequent procedures (e.g., first aid), including visual and auditory memories [36]. An unexpected finding in the present study was the reactivated traumatic memories of a mother who had also sustained a burn injury in her childhood. Therefore, future studies should consider parents’ past traumatic experiences. 

In this study, facing medical procedures was threatening and highly traumatic for both parents and children, mainly dressing changes. Witnessing the child’s procedural suffering and pain was a source of significant parental distress. McGarry et al. [35] corroborated and highlighted that the traumatic procedural reactions persist for up to six months post-burn, suggesting that some parents will relive the experience again, given the need to maintain post-discharge care (e.g., removing dressings in the bath). Egberts et al. [34] found similar results, but also noticed that these reactions during wound treatment decreased over time. In addition, a quantitative study found that invasive medical procedures were a significant predictor of parental PTSS over time [22]. In a qualitative study on parental experiences during the child’s wound care, parental pre-procedure emotional states appeared to be a key factor in their participation in treatment [34]. Parental emotional reactions should be considered, as recent evidence has shown that parents’ psychological adjustment (e.g., acute symptoms of distress) during the first dressing change impacts the child’s prognosis and clinical outcomes, such as delayed re-epithelialization, e.g., [46]. Despite that, our findings regarding parents’ perceptions of medical procedures also contradict the results reported in a previous qualitative study [36]. According to Egberts et al. [36], the medical procedures were not perceived as very threatening to parents since they were positively linked to the child’s recovery. In the present study, the results suggest that, although the parents recognized the importance of the treatments for the child’s recovery, they considered the treatment very threatening. Further research is needed to clarify these contradictory results in order to better understand the distressing nature of medical procedures and their implications on parents’ perceptions.

Another source of distress found was the observable changes in the child’s functioning, including mobility, appetite, skin, and sleep problems. A quantitative study found a positive association between parental distress and health-related problems (i.e., greater parental distress was associated with greater health-related problems), such as sleep problems and post-burn pruritus, in children under four years of age [47]. Changes in children’s functioning (e.g., mobility) and appearance have been associated with later social problems (e.g., bullying episodes and low social competence) [15], and most parents, when revisiting their experience in the hospital (e.g., when talking with researchers), were able to identify these specific burn-related outcomes [36], which suggests that this is an important target for intervention. This distress interacts with the parents’ most recurrent concern about the future, particularly the child experiencing physical or psychological sequelae as a result of the burn injury. These findings were consistent with previous qualitative studies, e.g., [33,35,48]. For example, Brown et al. [33] found that parents reported significant concerns regarding potential child physical sequelae (e.g., permanent scarring), and McGarry et al. [35] found that when the child returned to the community, dealing with the public stigma related to visible scars, was a challenging experience for parents. Regardless of the child’s age, most parents’ concerns were linked to scars and their implications (e.g., social problems) [48]. The development of scars has an uncontrollable and unpredictable nature, which produces parents’ feelings of helplessness [35]. Moreover, a study focusing on the burden of pediatric burns concluded that parents of pediatric burn survivors with visible scars had more difficulty coping with the physical sequelae of burn injuries than parents of survivors with hidden scars [32]. Thus, these concerns about an uncertain future must be addressed and discussed by the healthcare team with the parents, as suggested by other authors, e.g., [48]. In fact, parent-healthcare provider communication, a central component of family-centered care, is essential to increase satisfaction with the care provided and improve overall outcomes for both parents and children, particularly during the child’s medical procedures [33], as well as to prepare and negotiate the transition from the inpatient phase to post-discharge phase (e.g., home care) [49]. According to Brown et al. [33], healthcare providers should be prepared for parents’ emotional disclosures (e.g., distress) and communicate efficiently by providing parents with clear information about prevention, the steps of medical procedures, pain management, prognosis, and healing process, in order to relieve parental distress rather than contribute to parental uncertainty and concern. 

For parents, the child’s recovery was the most positive part of the whole experience. De Young et al. [22] also suggested that, as the child improves, parents will get better, especially since distressing medical procedures (which decrease in number as the child recovers) and child distress were considered potential risk factors for parental maladjustment. Furthermore, our findings showed that the child’s physical and psychological well-being was effectively ensured due to the quality of the healthcare and the hospital environment. Previous qualitative research was consistent with these findings, showing that the majority of parents reported having confidence in the healthcare team and satisfaction with the care provided to their children [36,49]. 

An original finding, in the present study, was the parent’s feelings of neglect during the hospital stay, reported as a loss of one’s identity, being reduced to “the child’s companion”. Future qualitative studies should explore the role of feelings of neglect during the hospital stay period and identify potential factors that may contribute to their development in order to better understand and satisfy parents’ needs.

Furthermore, in the present study, none of the parents received psychological support, and some parents were unaware of the hospital’s psychological services/resources. These findings, coupled with the unmet needs identified by parents (e.g., lack of support and opportunities for self-care), which is in line with previous qualitative studies, e.g., [28,30], raise an important contribution to healthcare research, in this area, underlining the need for parents’ psychological support and screening. The Integrative (Trajectory) Model of Pediatric Medical Traumatic Stress [50] provides a useful framework to guide psychosocial assessment and intervention for both pediatric patients and families, considering subjective responses/appraisals and offering specific intervention goals for each medical phase (i.e., peri-trauma, acute medical care, and ongoing care or discharge from care). According to this model, psychosocial screening and trauma-informed care should be provided at hospital admission [50]. In addition, regarding parents’ maladjustment outcomes, a recent study highlighted the pressing need to include caregivers in the burn survivors’ care plan starting at hospital admission [29]. 

The results of the present study support the need to develop and implement an integrated care plan geared toward the needs and suggestions (e.g., enhancing supportive care and environment) listed by parents of burn-injured children. In fact, pediatric burn centers must have a defined and adequate strategy to receive and support parents, preparing them for the post-discharge period [30]. In addition, healthcare provision should include a specialized multidisciplinary team in pediatric burns to improve clinical outcomes [15]. The results within the theme “finding ways to guide parents” are also relevant for informing future family-centered intervention programs to improve parent and child outcomes. An integrated family-centered care approach is paramount within the context of pediatric burn injuries [10,36]. Mohammadzadeh et al. [51] also encouraged the adoption of a family-centered empowerment model, given its role in promoting the quality of life of pediatric burn patients and reducing parental stress. A longitudinal qualitative study focusing on the psychosocial trajectories of parents of critically injured children highlighted the importance of early intervention and ongoing care, given the complex needs of these families and the persistence of parental distress, over time [52]. To ensure consistency and optimize the quality of the delivery of integrated healthcare to these families, the authors recommend a family support healthcare coordinator [52]. In this line, understanding and addressing parents’ healthcare needs and emotional and psychosocial adjustment difficulties is imperative to improve the health and psychological outcomes of both parents and burn-injured children. Therefore, multidisciplinary healthcare teams, including clinical and health psychologists, are crucial, and an empirical literature review found promising results regarding these practices within pediatric burn injury care [15]. Moreover, a recent case report in the field of burn care highlighted the roles and several benefits of mental health professionals in integrated care when families are involved (e.g., routine psychosocial screening) [53]. 

According to our graphical map (Figure 1), integrated care is paramount with intervention programs, including self-regulation training, active coping strategies, parent-healthcare provider communication, and family empowerment, in order to promote more adaptive outcomes and improve overall parents’ and children’s quality of life. 

Despite the strengths of this study, some limitations must be acknowledged. First, parents’ experiences were limited to a single hospital, and the majority of participants were mothers (82.6%), limiting the generalization of the results. Second, logbook weaknesses: although the open-ended questions captured a wide variety of information, some parents were very short in their answers. Finally, the present study did not consider the socio-economic and ethnic status of the parents, which may have an impact on the results. 

Future studies should include a balanced number of fathers and mothers, explore the views of healthcare providers, employ a mixed-method design, and control the effect of potential confounders (e.g., socio-economic variables).

## 5. Conclusions

This is the first qualitative study that addressed and explored the perceptions and experiences of Portuguese parents during the hospital stay of preschool children who had sustained an unintentional burn injury. Our qualitative findings showed that these parents were significantly burdened and distressed during this phase and considered that this process was typically more traumatic and challenging for them than for their children. In addition to raising several important clinical implications (e.g., establishing targeted goals for intervention), this study also adds new original data about the parents’ emotional and psychosocial adjustment and healthcare needs, such as the fact that most of these parents feel neglected (i.e., reduced to the role of the child’s companion) during the inpatient experience, and helped clarify previous contradictory findings regarding the perceived traumatic and distressing impact of medical procedures (e.g., dressing changes was the most negative aspect of the whole experience). Taken together, the qualitative findings suggest that there is a pressing need for integrated care in the field of pediatric burn injuries. In addition, parents should be screened early and included in the treatment care plans starting at the child’s hospital admission, in order to improve their adjustment, prevent the development of clinical traumatic symptoms, and also provide vital resources and strategies for the later stages of care (e.g., post-discharge care).

Finally, the present findings may be considered a first step to inform the development of family-centered intervention programs within integrated healthcare delivery. 

## Figures and Tables

**Table 1:** Parents’ sociodemographic and clinical characteristics (*n* = 46).

Parents’ Characteristics	*n* (%)	Mean (*SD*)	Range
Age	46	33.54 (5.82)	22–47
Gender			
Men	8 (17.4)		
Women	38 (82.6)		
Residential area			
Urban	30 (65.2)		
Rural	16 (34.8)		
Marital status			
Single	6 (13.0)		
Married	20 (43.5)		
Living with partner	19 (41.3)		
Divorced or separated	1 (2.2)		
Education			
With higher education	34 (73.9)		
Without higher education	12 (26.1)		
Employment status			
Inactive	15 (32.6)		
Active	31 (67.4)		
Chronic illness			
No	35 (76.1)		
Yes	11 (23.9)		
Medication			
No	33 (71.7)		
Yes	13 (28.3)		
Witnessed the burn event			
No	21 (45.7)		
Yes	25 (54.3)		

**Table 2:** Children’s sociodemographic and clinical characteristics (*n* = 46).

Children’s Characteristics	*n* (%)	Mean (*SD*)	Range
Age (in years)	46	2.28 (1.52)	0.67–6
Gender			
Boys	28 (60.9)		
Girls	18 (39.1)		
Number of siblings	27	1.44 (0.80)	1–4
Enrollment in preschool			
No	21 (45.7)		
Yes	25 (54.3)		
Pre-existing medical conditions			
No	45 (97.8)		
Yes	1 (2.2)		
Place where the burn occurred			
At home	44 (95.7)		
Outside the home	2 (4.3)		
Mechanism of burn			
Scald	34 (73.9)		
Contact	4 (8.7)		
Flame	3 (6.5)		
Friction	2 (4.3)		
Other (e.g., chemical)	3 (6.5)		
Number of burn regions sustained			
Single	13 (28.3)		
Multiple	33 (71.7)		
Burn depth			
Superficial partial-thickness (2nd degree)	6 (13.0)		
Deep partial-thickness (2nd degree)	31 (67.4)		
Full-thickness (3rd degree)	9 (19.6)		
Visible burns ^a^			
No	22 (47.8)		
Yes	24 (52.2)		
Edema			
No	13 (28.3)		
Yes: Local edema	33 (71.7)		
Edema texture			
Soft edema	31 (67.4)		
Hard edema	2 (4.3)		
Skin graft			
No	7 (15.2)		
Planned	39 (84.8)		
Hospital length of stay			
Less than 2 weeks	8 (17.4)		
2 weeks or more	38 (82.6)		
Days since burn injury	46	6.26 (3.09)	1–17
%TBSA	46	4.71 (3.10)	1–13

Note. %TBSA = percentage of the total body surface area burned. ^a^ Burn injuries affecting the hands, neck, face, and/or head.

**Table 3:** Themes, sub-themes, and representative quotes from the participants’ records.

Themes	Sub-Themes (*n*, %)	Examples
Diving into the crisis of the child burn injury	Negative trauma responses (44, 95.65%)	“*I feel very bad (…) I feel that they look at us like we’re guilty*.” (mother of a two-year-old boy)
	Facing medical procedures (34, 73.91%)	“*It was very distressing to see my daughter screaming in pain during the dressing change…*” (mother of a ten-month-old girl)
	Changes in the child’s functioning (27, 58.70%)	“*Because of the bandages on his hands, he can’t do some of the activities he is used to, such as eating, playing with other children…*” (mother of a one-year-old boy)
	COVID-19 stressors (33, 71.73%)	“*(…) a bit apprehensive, afraid that he might get COVID again because that slows down the whole process*.” (mother of a five-year-old boy)
	Unmet needs (38, 82.61%)	“*I miss my family and the support they could give me, for example, here I don’t have anyone to help me feed my daughter. (…) It is in these situations that we see that we’re just the child’s companions…*” (mother of a nine-month-old girl)
Being together and in good hands	Child’s well-being and recovery (34, 73.91%)	“*The day my daughter (with a facial burn) opened her eyes again was a victory*.” (father of a ten-month-old girl)
	Quality of healthcare and environment (35, 76.09%)	“*The medical team made me feel confident (…) The people here in the hospital talk a lot about the experience they’ve had/are having, and that helped me a lot to take it all in*.” (mother of a one-year-old boy)
Becoming aware of an uncertain future	Ongoing suffering of the child and (potential) permanent scaring (41, 89.13%)	“*I am afraid there will be some rejection from him or his classmates or even bullying episodes. I hope he doesn’t have any physical or emotional marks*.” (mother of a six-year-old boy)
	Concerns about being blamed and that a burn might happen again (8, 17.39%)	“*(…) that my daughter blames me when she looks at the scars*.” (mother of a one-year-old girl)
Enhancing supportive care and environment	Strengthening support and adapting the care plan (29, 63.04%)	“*The food should be more appropriate for children of this age, and we would have more support in this regard, such as helping to feed them, distracting them while they eat, etc*.” (father of a three-year-old boy)
	Improvement of hospital facilities (8, 17.39%)	“*We should only have single rooms so that families can have their privacy and feel at ease*.” (mother of an 11-month-old boy)
Finding ways to guide parents	Maintaining a positive mindset and activating coping resources (41, 89.13%)	“*Have hope that better days will come*.” (mother of a one-year-old girl)
	Recognizing parental distress and promoting a child’s well-being (21, 45.65%)	“*This turns out to be more difficult for us than for them*.” (mother of a three-year-old boy)
	Raising awareness (9, 19.57%)	“*People should talk more about this issue of burns, because it happens a lot, especially in children, and you don’t hear about it*.” (mother of a one-year-old boy)

## Data Availability

For ethical and privacy reasons, the data are not publicly available. The data can be made available upon reasonable request to the corresponding author.
